# Association of astrocytes with neurons and astrocytes derived from distinct progenitor domains in the subpallium

**DOI:** 10.1038/srep12258

**Published:** 2015-07-20

**Authors:** Makio Torigoe, Kenta Yamauchi, Yan Zhu, Hiroaki Kobayashi, Fujio Murakami

**Affiliations:** 1 Graduate School of Frontier Biosciences, Osaka University, Suita, Osaka 560-8531, Japan

## Abstract

Astrocytes play pivotal roles in metabolism and homeostasis as well as in neural development and function in a manner thought to depend on their region-specific diversity. In the mouse spinal cord, astrocytes and neurons, which are derived from a common progenitor domain (PD) and controlled by common PD-specific transcription factors, migrate radially and share their final positions. However, whether astrocytes can only interact with neurons from common PDs in the brain remains unknown. Here, we focused on subpallium-derived cells, because the subpallium generates neurons that show a diverse mode of migration. We tracked their fate by *in utero* electroporation of plasmids that allow for chromosomal integration of transgenes or of a *Cre recombinase* expression vector to reporter mice. We also used an *Nkx2.1*^*Cre*^ mouse line to fate map the cells originating from the medial ganglionic eminence and preoptic area. We find that although neurons and astrocytes are labeled in various regions, only neurons are labeled in the neocortex, hippocampus and olfactory bulb. Furthermore, we find astrocytes derived from an *Nkx 2.1*-negative PD are associated with neurons from the *Nkx2.1*^*+*^ PD. Thus, forebrain astrocytes can associate with neurons as well as astrocytes derived from a distinct PD.

Astrocytes constitute the most abundant cell type in the CNS and play important roles in the regulation of blood flow, maintenance of the cerebrospinal fluid, ion, pH and metabolism^1^. In addition, astrocytes play critical roles in neural development and functions^2^,^3^,^4^,^5^,^6^,^7^. Given that they possess a region-specific heterogeneity^8^,^9^, this heterogeneity may be important for interactions between astrocytes and neurons that differentiated in a region-specific manner. Indeed, in the spinal cord astrocytes originating from the motor neuron (MN) PD, but not from other PDs along the dorsoventral axis, are involved in synaptogenesis on MNs^4^. Similarly, a repellent expressed in ventral spinal cord astrocytes regulates the axon growth orientation of the ventral spinal cord MNs^7^. These findings raise the possibility that the common developmental history between astrocytes and neurons are important for their interactions.

Studies in spinal cord and cortical astrocyte development are consistent with this notion. Transcription factors expressed segmentally along the dorsoventral axis and control neuronal subtypes, also regulate the segment-specific molecular phenotype of astrocytes^10^,^11^. In the neocortex, neurons and astrocytes located in the same radial column are generated from common progenitors^12^,^13^,^14^. Given that both neurons and astrocytes migrate radially in these regions^4^,^13^,^14^, neurons and astrocytes with a common PD may settle to the same region, allowing them to interact with each other.

In the forebrain, there are neurons migrating through tortuous pathways^15^,^16^,^17^,^18^, implying that such neurons may not localize near astrocytes with a common PD. However, co-localization of neurons and astrocytes from distinct progenitor domains has not been reported. To address this issue, we labeled progenitors in the subpallium using *in utero* electroporation (IUE). The subpallium was chosen, because it is a source of various types of forebrain neurons^19^,^20^, which show a diverse mode of migration^15^,^17^. To label all descendants from ventricular zone progenitors including glial cells, we utilized the Tol-2 transposon system (hereafter Tol-2 system)^21^,^22^ or IUE of a *Cre* expression vector^23^ to *Ai9* reporter mice. We also used *Nkx2.1*^*Cre*^ mice to fate map the progenitors in specific subpallial PDs. We find while both neurons and astrocytes are labeled in some regions, only neurons are labeled in some other regions, implying that they are associated with astrocytes from a distinct domain. Indeed, we find that astrocytes and neurons originating from distinct PDs are closely associated in the striatum, olfactory tubercle and piriform cortex. Incidentally, we also find astrocytes associated with those from a distinct domain.

## Results

### Site of labeling

It is not possible to determine the exact site of IUE by observing labeled brain in mature animals. However, examination of embryos soon after IUE would allow us to predict the site of IUE. Therefore, several embryos were fixed 2–4 days after electroporation (6 embryos at E12.5, 2 at E13.5 and 1 at E14.5) and labeled sites were examined (Supplementary Figure S1). The lateral ganglionic eminence (LGE) was labeled in all 9 samples (Supplementary Figure S1A-B) with the ventral pallium labeled in 4 (data not shown) and the medial ganglionic eminence (MGE) in the remaining 5 samples (Supplementary Figure S1B) (Table 1). The caudal ganglionic eminence (CGE) was labeled in 7 samples (Supplementary Figure S1C). These findings indicate that the site of IUE was mostly ganglionic eminences (GEs), although the LGE and CGE tended to be preferentially labeled.

### IUE caused labeled cells in various forebrain structures

In theory IUE using Tol-2 system or *Cre* expression vectors combined with reporter mice enables us to label all descendants derived from electroporated progenitors. Indeed, we previously showed that this is the case in the cerebellum^24^.

#### Only neurons were labeled in the neocortex, hippocampus and olfactory bulb

**Figure 1:**
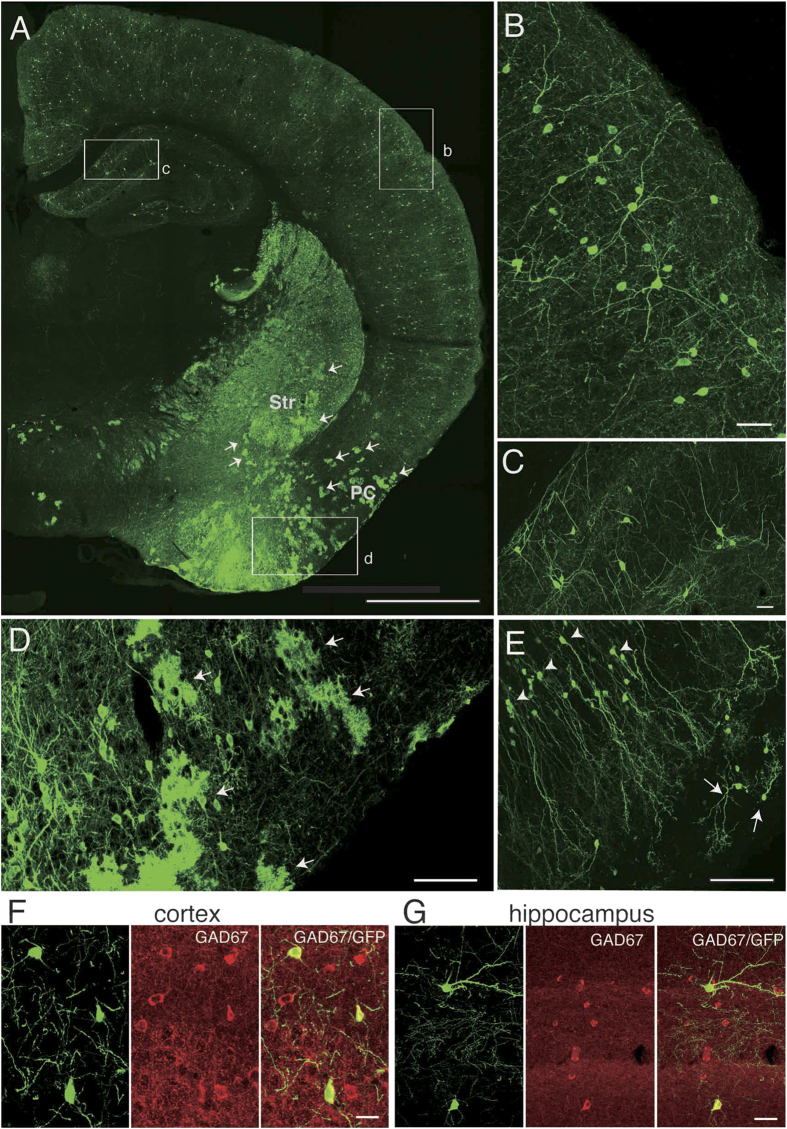
IUE labeled only neurons in the neocortex, hippocampus and olfactory bulb but also labeled astrocyte-like cells in the subpallium. **A**, Coronal section at the level that includes the striatum and hippocampus. Arrows point to cloud-like structures in the striatum (Str) and the piriform cortex (PC), which were identified as astrocytes using high-resolution views (see Fig. 2). **B**, High-magnification view of the area shown by the rectangle labeled “b” in A. Cells with neuronal morphology are labeled in the neocortex. **C**, High-magnification view of the area shown by the rectangle labeled as “c” in A, showing labeled cells with neuronal morphology in the hippocampus. Note the absence of cloud-like profiles either in the neocortex or the hippocampus. **D**, High-magnification view of the area shown by the rectangle “d” in A. Many cloud-like cells that appear to be astrocytes are labeled in the piriform cortex (arrows). **E**, Granule cells (arrowheads) and periglomerular cells (arrows) in the olfactory bulb. No astrocyte-like cells can be seen. **F**, **G**, GABAergic nature of labeled neurons in the neocortex (F) and hippocampus (G). Left panels in F and G show GFP-labeled cells, middle panels GAD67 immunolabeled cells and right panels merged views. Scale bars: 1 mm in A, 40 μm in B and C, 100 μm in D and E, and 20 μm in F and G.

It is well established that cortical interneurons originate from the MGE, CGE and preoptic area (POA) and tangentially migrate to their final destinations^25^,^26^,^27^. Indeed, IUE labeled many cells in the neocortex (Fig. 1A). The labeled cells were readily recognized as neurons based on their morphological features: 1) a round or ellipsoid-shaped cell body with smooth surface and 2) thick and tapering primary dendrites (see Fig. 1B,F). They were dispersed throughout the neocortex and showed varying morphology, from multipolar to bipolar shaped (Fig. 1B and data not shown), suggesting that they are interneurons. These cells expressed glutamate decarboxylase 67 (GAD67), a rate-limiting enzyme for the synthesis of GABA (Fig. 1F), indicating that these cells are indeed GABAergic interneurons. We did not observe labeling of pyramidal cells, which are the major type of excitatory neurons in the neocortex.

Inhibitory interneurons in the hippocampus also originate from the MGE and CGE and migrate tangentially^28^,^29^. Consistently, we also observed labeled cells in the hippocampus. These cells appeared to be interneurons due to the reasons described above for cortical interneurons (see Fig. 1C,G). Labeling of pyramidal cells, the major type of excitatory neurons in the hippocampus, was not observed.

Olfactory bulb interneurons originate from the LGE and migrate along the rostral migratory stream tangentially^19^,^30^,^31^. In the olfactory bulb, we observed neurons that were readily recognizable as granule cells based on their morphology (Fig. 1E, arrowheads). In addition, neurons that exhibit morphological features of periglomerular cells were also labeled (Fig. 1E, arrows). However, we did not observe labeling of mitral cells, which are the major type of excitatory neurons in the olfactory bulb.

The fact that neurons were exclusively labeled in the neocortex, hippocampus and olfactory bulb implies that the neurons in these regions are associated with astrocytes with distinct origins.

#### Both neurons and astrocytes were labeled in several subpallial regions

The cells in the piriform cortex originate from the dorsal LGE and corticostriatal border, and radially migrate towards the pia followed by a spread into the anteroposterior axis of the brain by tangential migration^32^,^33^,^34^. Neurons in the nucleus accumbens originate from the LGE^19^, although the exact route of their migration is unknown. Striatal neurons mostly originate from the MGE, LGE and CGE^19^,^20^. Neurons in the olfactory tubercle appear to originate from the ventral^34^ or dorsal LGE^32^ and spread after radial migration. Amygdalar neurons originate from the LGE, CGE, MGE, ventral pallium^19^,^20^,^35^,^36^ and POA^37^ in a region-specific manner^35^. At least a subset of amygdalar neurons appears to migrate radially along the lateral cortical stream^33^.

**Figure 2:**
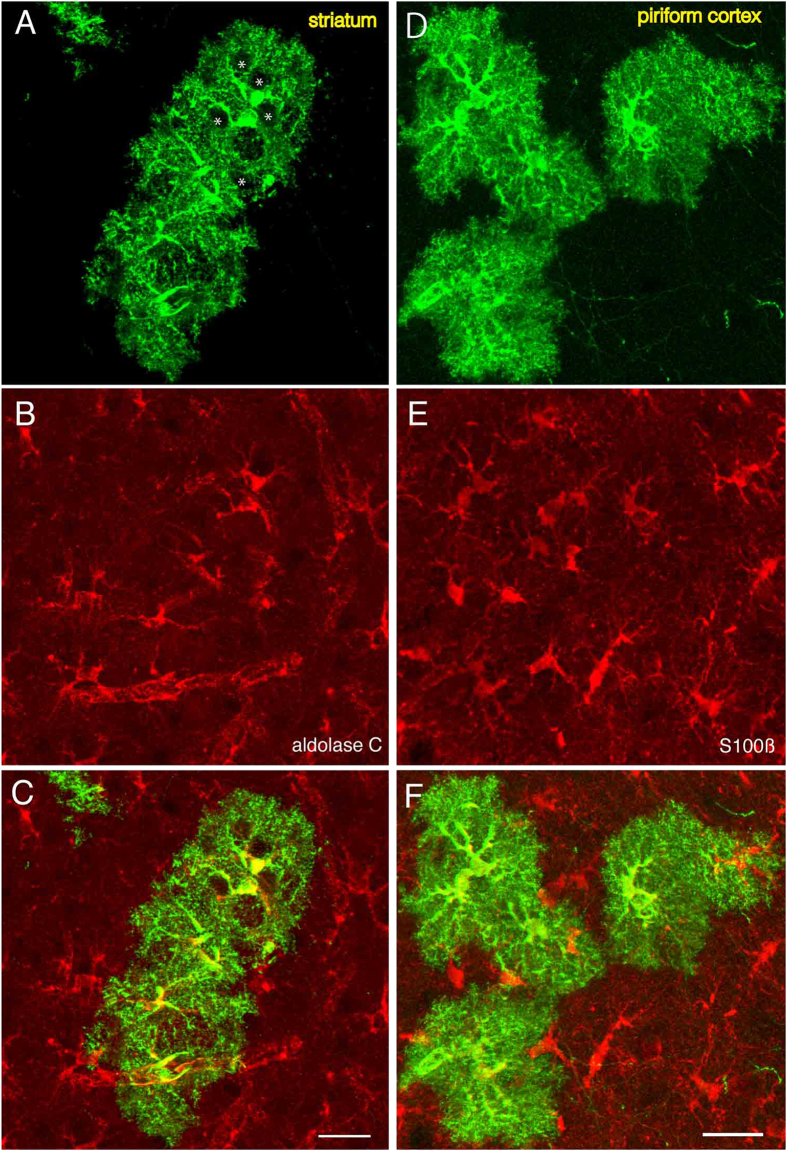
Identification of astrocytes by molecular markers. Astrocytes in the striatum (**A–C**) and piriform cortex (**D–F**). **A, D**, Astrocytes labeled by Tol2-GFP in the striatum and piriform cortex, respectively. Hole-like structure can be seen (A, asterisks). These structures are likely to be occupied by neurons (see below). B, Immmunostaining for aldolase **C. E**, Immunostaining for S100 beta. C and F are merged views of A and B, and D and E, respectively. Scale bars: 25 μm. The bar in C applies to A–C and that in F applies to D–F.

Consistent with these previous studies, we observed labeled cells in the nucleus accumbens, olfactory tubercle, striatum, amygdala and piriform cortex. Unlike in the neocortex, hippocampus and olfactory bulb, cells with cloud-like morphologies were often observed at low magnification (see Fig. 1A, arrows). Observation at a higher magnification revealed that these morphologies are due to extensively ramified processes by the cells (Fig. 1D). Figure 2 shows a high magnification view of such cells in the striatum (Fig. 2A–C) and piriform cortex (Fig. 2D–F). At this magnification it can be clearly seen that these cells have highly ramified morphologies with several thick and short branches (Fig. 2A,D), morphologies similar to those of protoplasmic astrocytes described previously^38^. The astrocyte nature of these cells was further examined by immunostaining against astrocyte markers. The cell bodies as well as the proximal portion of their processes were immunopositive for aldolase C^4^,^39^ (Fig. 2B) and S100 beta^40^ (Fig. 2E), confirming these cells are astrocytes.

In the piriform cortex, many neurons were labeled. In addition, many astrocytes, which were characterized by a cloud-like morphology at a low magnification, were also labeled (Fig. 1A, arrows). At high magnification, these cells showed extensively ramified processes of various calibers (Fig. 1D, see Fig. 2D also).

**Figure 3:**
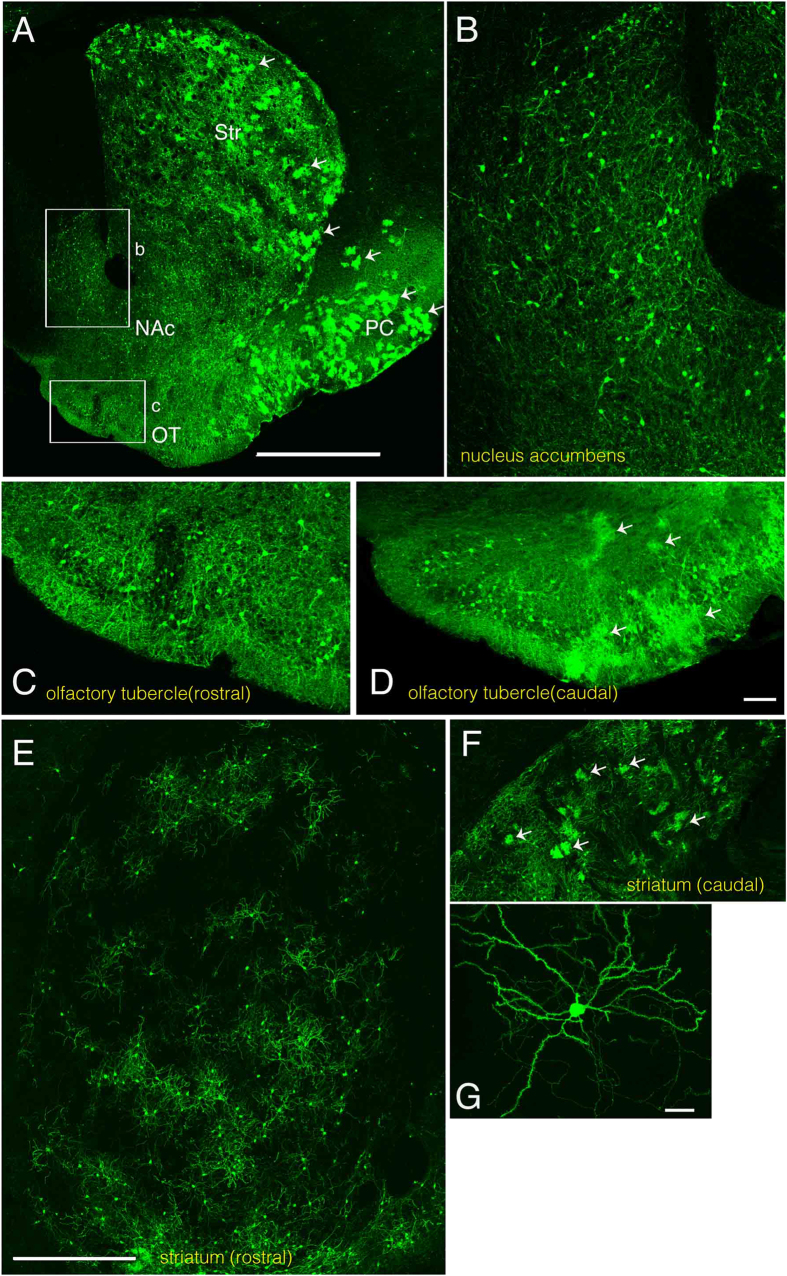
IUE-labeled neurons and astrocytes in the nucleus accumbens, olfactory tubercle, and striatum. **A**, Low-magnification image showing labeled striatum (Str), nucleus accumbens (NAc), olfactory tubercle (OT) and piriform cortex (PC). Many astrocytes are labeled in the striatum and the piriform cortex (arrows). Lateral is to the right and ventral is to the bottom. **B**,**C**, High magnification views of the boxed areas labeled “b” and “c” in A show regions of the nucleus accumbens shell region and olfactory tubercle, respectively. **D**, Olfactory tubercle at a caudal level of the same animal. While no cloud-like profiles can be detected at the rostral level (C), there are many at the caudal level (D), indicating an abundance of astrocytes there. In some embryos, the rostral striatum was almost exclusively occupied by neurons (E). In the caudal striatum, both neurons and astrocytes are also labeled (F)(arrows). **G**, Most labeled neurons in the striatum are medium spiny neurons. In E-G, lateral is to the left and ventral is to the bottom. Scale bars: 1 mm in A, 100 μm in D, 500 μm in E and 20 μm in G. The bar in E also applies to F.

We found the nucleus accumbens was mostly occupied by neurons (Fig. 3A,B). In the olfactory tubercle, both neurons and astrocytes were labeled. However, the rostral part was generally devoid of astrocytes (Fig. 3C), while the caudal part was occupied by both neurons and astrocytes (Fig. 3D arrows).

In the striatum, labeled neurons were often distributed throughout the structure. In addition to neurons, astrocytes were also labeled. However, the distribution of astrocytes was not uniform in some preparations. While the rostral striatum was almost devoid of astrocytes (Fig. 3E,G), astrocytes were dispersed among neurons in the caudal striatum of the same animal (Fig. 3F arrows).

**Figure 4:**
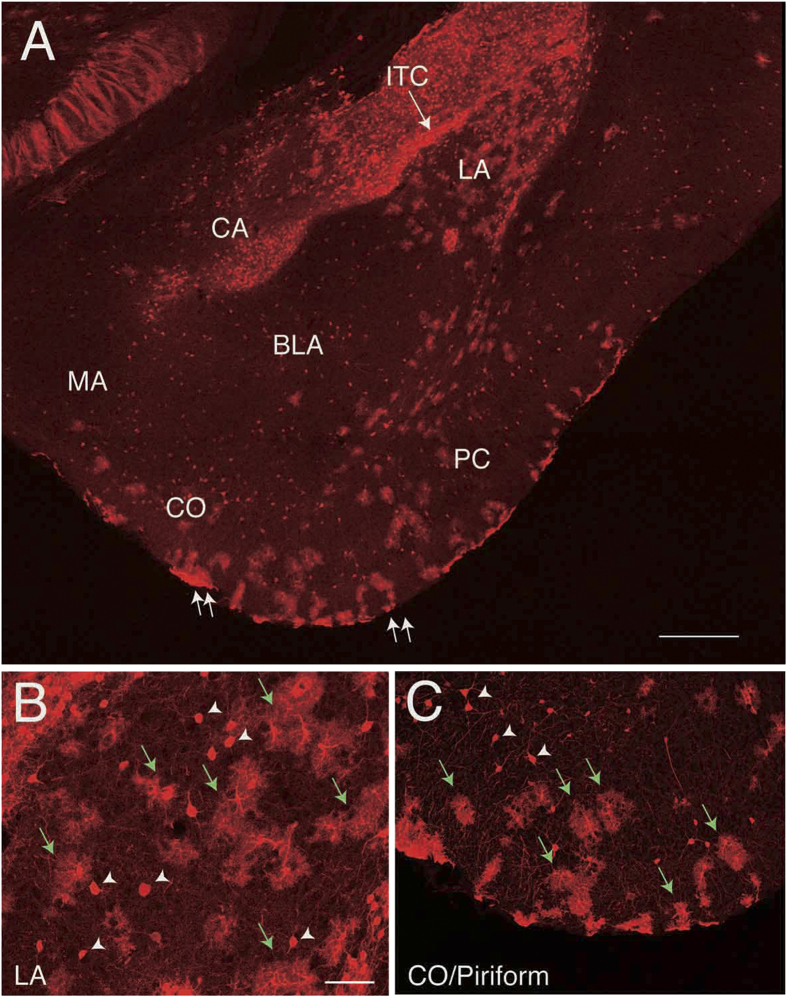
Labeled cells in the amygdala. **A**, Coronal section showing labeled neurons and astrocytes in the amygdaloid complex of an *Ai9* mouse electroporated with *Cre recombinase* expression vectors. Cells in the central amygdala (CA), lateral amygdala (LA), and cortical amygdala (CO) as well as the piriform cortex (PC) are labeled. Those in the basolateral amygdala (BLA) are sparsely labeled. Cells in the intercalated cell masses (ITC) are also labeled. In the LA there are both cloud-like cells and cells with clear contours indicating both neurons and astrocytes are labeled, while in the CA there are no cloud-like cells, indicating only neurons are exclusively labeled. **B**, High magnification view of the LA. **C**, High magnification view of the area encompassing the CO and the PC (between the two double arrows). In addition to neurons (arrowheads), astrocytes are labeled (green arrows). Scale bars: 500 μm in A. and 50 μm in B. The bar in B applies to C but is 100 μm.

In the amygdala, both neurons and astrocytes were labeled. The amygdala is composed of a dozen of nuclei, but here we focused on nuclei that can be easily recognized on a coronal section that includes both lateral (LA) and basolateral nuclei (BLA) (Fig. 4). In this plane of section, nuclei of the medial amygdala (MA), central amygdala (CA) and cortical amygdala (CO) were also included. Neurons of all of these nuclei could be labeled, although only subsets of these nuclei were labeled in some preparations. We also observed labeling of astrocytes (green arrows in Fig. 4B–C). Although the regions occupied by astrocytes largely coincided with those of neurons, the MA (data not shown) and CA (Fig. 4A) were sometimes only occupied by neurons.

In summary, IUE in the present study exclusively labeled interneurons in the neocortex, hippocampus and olfactory bulb, while both neurons and astrocytes were labeled in various subpallial structures. These findings suggest that neurons and astrocytes derived from common PDs do not necessarily share the region of distribution in mature brain.

### Labeling of astrocytes in Nkx2.1^Cre^; Ai9 mice

In IUE-labeled preparations, the extent and the pattern of astrocyte labeling varied from sample to sample. For example, while no astrocytes were labeled in the rostral striatum in some samples, many were labeled in other samples (data not shown). This is probably due to the variation of labeled site among different samples as shown in Supplementary Figure S1 and Table 1. We therefore decided to analyze *Nkx2.1*^*Cre*^*; Ai9* mice to examine the relationship between a defined PD and their descendants.

Nkx2.1 is a transcription factor expressed in most part of the MGE and POA of embryonic brains (see Supplementary Figure S1D–F). A previous genetic fate-mapping study using *Nkx2.1*^*Cre*^*; Z/EG* mice showed that *Nkx2.1*^*+*^ PD gives rise to a subset of neurons in the olfactory bulb, neocortex, hippocampus, striatum, amygdala, lateral septal nuclei, diagonal band of Broca and olfactory tubercle^41^.

**Figure 5:**
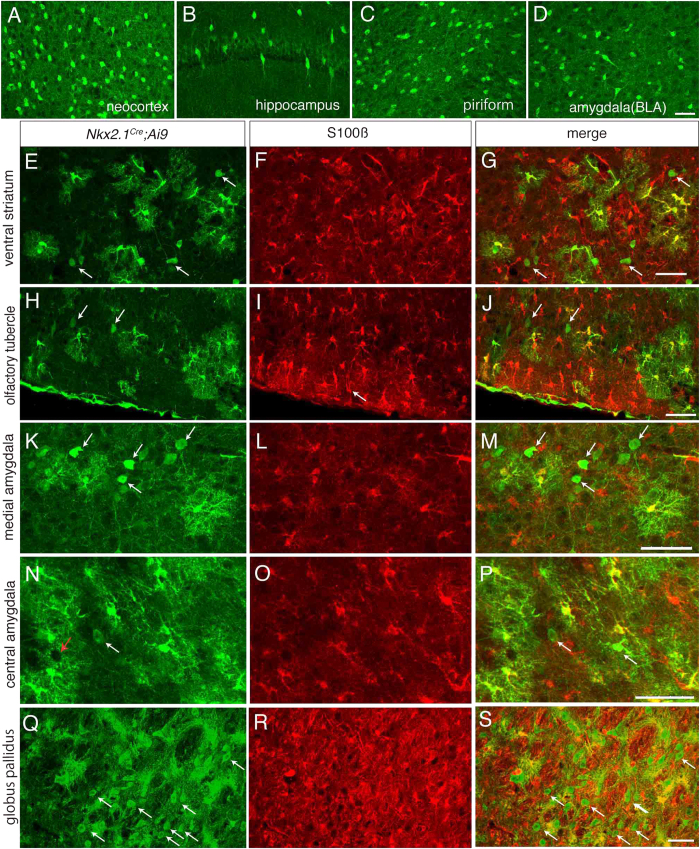
Labeled neurons and astrocytes in *Nkx2.1*^*Cre*^*; Ai9* mice. A–D, Labeled cells in the neocortex (**A**), hippocampus (**B**), piriform cortex (**C**) and basolateral amygdala (**D**). None of the labeled profiles have ramified processes, suggesting that they are not astrocytes. **E–S**, Neurons and astrocytes labeled in the ventral striatum (**E,G**), olfactory tubercle (**H,J**), medial amygdala (**K,M**), central amygdala (**N,P**) and globus pallidus (**Q,S**). Unlike **A–D**, cells with ramified processes are intermingled with neuron-like cells (arrows in **E,H,K,N,Q,G,J,M,P** and **S**). The former are positive for S100 beta (**F,I,L,O,R**), an astrocyte marker. G,J,M,P and S are merged views showing double labeled cells in yellow. Red arrow in N points to a hole-like structure. In A,B,C,D,E,H,K,N and Q, red signal of tdTomato was converted to green. Scale bar: 50 μm for all panels. The bar in D applies to A–D, that in G to E–G, that in J to H–J, that in M to K–M, that in P to N–P and that in S to Q–S.

We first confirmed that tdTomato signal was prominently expressed in the MGE except the dorsalmost region and the POA in E12.5 *Nkx2.1*^*Cre*^*; Ai9* mouse embryos, while neither LGE or CGE expressed tdTomato (Supplementary Figure S1F). We then observed mature animals and found that labeled cells in the neocortex, hippocampus, striatum, globus pallidus, amygdala, olfactory tubercle, nucleus accumbens, lateral septal nuclei, diagonal band of Broca and piriform cortex (see Fig. 5 and Supplementary Figure S2), consistent with the previous report^41^. Most of these cells exhibited a round or ovoid shape and smooth surface, sometimes extending a few, usually unbranched processes, suggesting that they are neurons (Fig. 5A–D and arrows in Fig. 5E,H,K,N,Q). Indeed, they were immunopositive for a neuronal marker, NeuN (Supplementary Figure S2B-E), but negative for an astrocyte marker, aldolase C (Supplementary Figure S3).

We noticed that there was also a second type of labeled cells in *Nkx2.1*^*Cre*^*; Ai9* mice. These cells extended thin ramified processes from the cell body with irregular shape, a morphological feature typical with astrocytes. Although we hardly found astrocytes in the neocortex (Fig. 5A), hippocampus (Fig. 5B), piriform cortex (Fig. 5C) or most regions of the amygdala (Fig. 5D), we did find astrocytes in the ventral part of the striatum and nucleus accumbens, albeit sparsely (Fig. 5E–G). In addition, astrocytes were densely labeled in the olfactory tubercle (Fig. 5H–J), globus pallidus (Fig. 5Q–S) and lateral septal nucleus (data not shown). In the amygdala, we also found labeled astrocytes, although their distribution was restricted to the MA (Fig. 5K–M) and CA (Fig. 5N–P).

In summary, although *Nkx2.1* progenitors generate both neurons and astrocytes, they generate astrocytes only in restricted regions, supporting the notion that neurons and astrocytes derived from a common PD do not necessarily share the region of distribution.

### Association of neurons and astrocytes from distinct domains

In the current study, IUE tended to preferentially label progenitors in the LGE and CGE (Table 1), where *Nkx2.1* is not expressed^42^. Moreover, we noticed that IUE-labeled cortical neurons in mature brains are often distributed in the superficial layers of the neocortex (data not shown), a feature characteristic to cortical interneurons of CGE origin^43^,^44^,^45^. This finding is consistent with the interpretation that IUE in our study tended to label astrocytes originating from PDs other than *Nkx2.1*^*+*^PDs.

**Figure 6:**
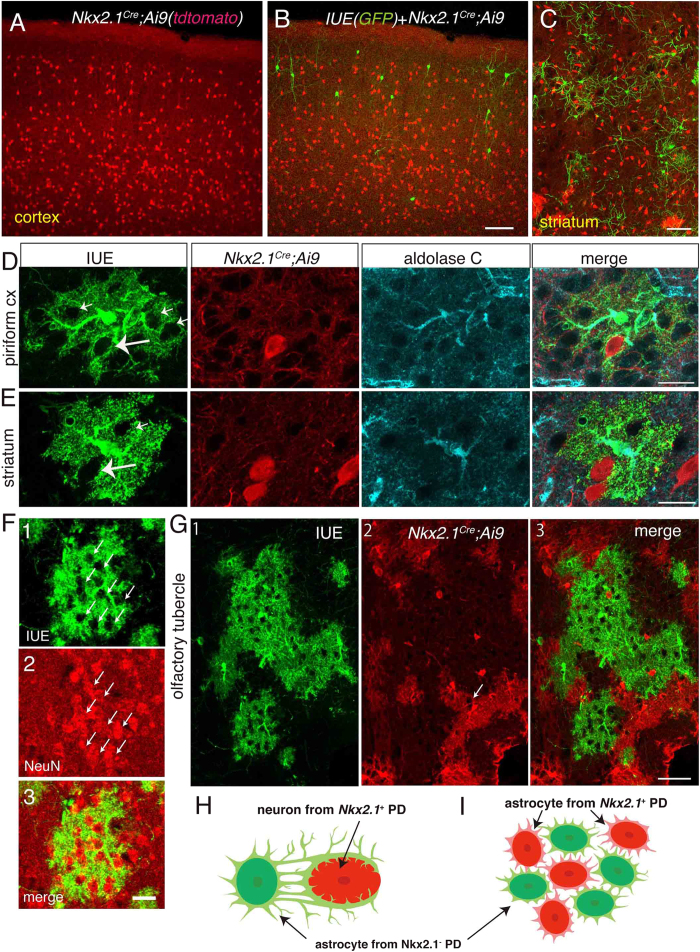
Coexistence of neurons and astrocytes from distinct origins. IUE was performed on *Nkx2.1*^*Cre*^*; Ai9* animals. **A, B**, In the neocortex, IUE-labeled GFP^+^ neurons (green) are distributed in upper layers where tdTomato^+^ cells from *Nkx2.1*^*+*^ PD (red) (**A**) are distributed only sparsely. There is no co-localization of green and red signals (**B**), indicating distinct origins of the red and green cells. Although the distribution of GFP^+^ cells and tdTomato^+^ cells are different, both cells coexisted in some regions such as the neocortex (**B**) and striatum (**C**). In regions such as the piriform cortex (**D**) and striatum (**E**), we occasionally encountered GFP^+^ astrocytes in association with tdTomato^+^ neurons (leftmost panels of D and E). F, Astrocytes enwrapping neurons. Large white arrows point to hole-like structures occupied tdTomato^+^ neuron. There are also hole-like structured not occupied by tdTomato-labeled neurons (small arrows). **F1**, IUE-labeled GFP^+^ astrocyte. **F2,** Immunostaining for NeuN, showing most hole-like structures in F1 (arrows) are occupied by neurons (F2). **F3**, Merged view. These neurons are tdTomato^-^, indicating that they originated from PDs other than *Nkx2.1*^*+*^ PD. **G**, IUE-labeled GFP^+^ astrocytes (**G1**) and those from *Nkx2.1*^*+*^ PD (G2) in the olfactory tubercle. **G3** is a merged view. In this region, intermingled distribution of tdTomato^+^ and GFP^+^ astrocytes was observed. However, there is no overlap of their processes. White arrow in **G2** points to a hole-like structure. **H**, Schematic illustration of a neuron from the *Nkx2.1*^+^ PD (red) is contacted by an astrocyte from a distinct PD (green). **I**, Schematic illustrating astrocytes from two distinct PDs are associated, intermingled without invading the territory of those from other PDs. Astrocytes with different colors represent those from distinct PDs. Scale bars: 100 μm in A–C, 20 μm in D, E and F, and, 50 μm in G.

We thought that this would open a possibility to examine the relationship between labeled neurons and astrocytes from distinct PDs in the same animal and performed IUE to *Nkx2.1*^*Cre*^*; Ai9 mice*. In animals in which IUE with the Tol-2 system was performed on *Nkx2.1*^*Cre*^*; Ai9* mice (n = 5), we found GFP-labeled cells in the neocortex, hippocampus, striatum, piriform cortex and amygdala, but none of these cells expressed tdTomato (Fig. 6) (n = 4/5), indicating that they originated from *Nkx2.1*-negative PDs. This is illustrated in Fig. 6A,B, which show labeled neurons in the neocortex. Although GFP^+^ cells were partially intermingled with tdTomato^+^ cells, none of them co-expressed tdTomato (Fig. 6B). This was also the case in other regions of the brain. Although GFP^+^ cells and tdTomato^+^ cells were intermingled with each other in the ventral part of the striatum (Fig. 6C), none of the labeled cells co-expressed GFP and tdTomato. In one animal (n = 1/5), a few cells co-expressed tdTomato and GFP, although a vast majority of labeled cells were either GFP or tdTomato single positive. These results indicate that IUE-labeled cells are almost completely distinct from *Nkx2.1* progenitor descendants in these animals.

Successful labeling of descendants from two distinct PDs in the same animal allowed us to examine the spatial relationship between neurons and astrocytes from distinct PDs. Although labeled regions from *Nkx2.1* PD were largely segregated from those by IUE particularly for astrocytes, we occasionally found neurons and astrocytes from distinct regions were closely associated in the striatum, olfactory tubercle and piriform cortex. Figure 6D and E show tdTomato-labeled neurons in *Nkx2.1*^*Cre*^*; Ai9* animals and IUE-labeled GFP^+^ astrocytes in the piriform cortex and striatum, respectively. The somata of tdTomato-labeled cells were enwrapped by the processes of GFP^+^ astrocytes (rightmost panels of Fig. 6D,E, see Supplementary Figure S4 also). Similar results were obtained in all animals (n = 5/5), including the one in which a small number of neurons were co-labeled by tdTomato and GFP. Furthermore, we found astrocytes displaying hole-like structures that were not filled by its processes (Fig. 6D). Most of these structures were not occupied by either GFP^+^ or tdTomato^+^ neurons (Fig. 6 small arrows), but NeuN-positive (Fig. 6F). Together, these results suggest the occurrence of contacts between astrocytes and neurons from distinct PDs (Fig. 6H).

In the spinal cord of the mouse, domain-specific astrocytes along the dorsoventral axis are strictly regulated and invasion from the neighboring region does not take place even after depletion of astrocytes in a specific domain^4^. However, we noticed that astrocytes originating from the *Nkx2.1*^*+*^ PD were intermingled with those from *Nkx2.1*^–^ PDs in *Nkx2.1*^*Cre*^*; Ai9* mice (Fig. 5E–S). Indeed, examination of IUE-labeled *Nkx2.1*^*Cre*^*; Ai9* mice indicated that in some regions of the piriform cortex, striatum and olfactory tubercle, tdTomato^+^ astrocytes were intermingled with GFP^+^ astrocytes, although the distributions of these two types of astrocytes were largely complementary. Curiously, we sometimes encountered one of these differently labeled astrocytes adjacent to one another, and sometimes even seemingly in direct contact (Fig. 6G). It is important to note that labeled astrocytes never co-expressed tdTomato and GFP. These astrocytes from distinct PDs did not share spatial domain of existence (Fig. 6G merge), a relationship reminiscent of that of neighboring astrocytes in the hippocampus^46^. Thus, although astrocytes from distinct PDs can contact with each other, they do not violate the spatial domain of astrocytes from other PDs (Fig. 6I). Similar results were obtained in 4 animals (see Supplementary Figure S4). In one animal, the density of IUE-labeled astrocytes was too low to examine this type of relationship. Collectively, these findings suggest that astrocytes from distinct PDs interact each other at least in some regions in the subpallium.

## Discussion

**Figure 7:**
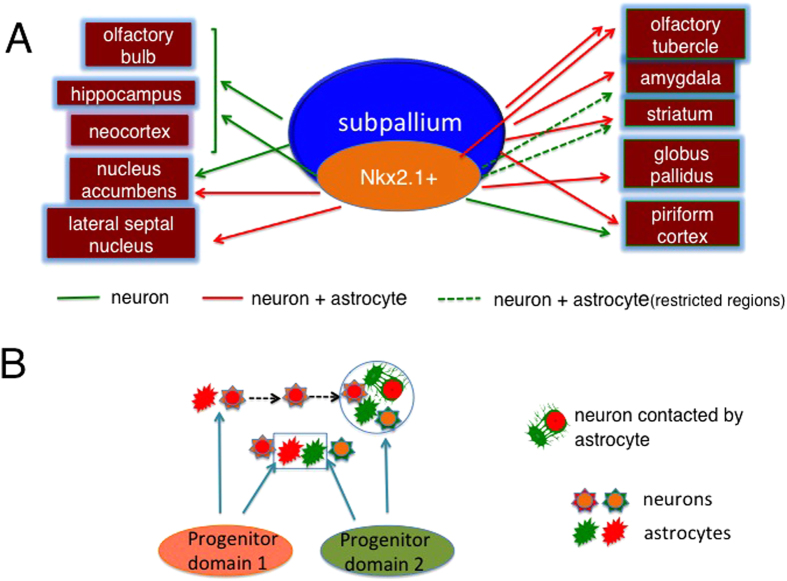
Schematics summarizing the results of the present study. **A**, Labeling of progenitors in the subpallium by IUE caused the labeling of neurons in the neocortex, hippocampus, olfactory bulb and nucleus accumbens (green arrows), but the labeling of both neurons and astrocytes in the olfactory tubercle, amygdala, striatum, globus pallidus and piriform neocortex (red arrows). Dotted green arrows represent the supply of astrocytes to a restricted region within the indicated structure. Fate-mapping using *Nkx2.1*^*Cre*^*; Ai9* mice caused the labeling of only neurons even in the piriform neocortex, dorsal striatum and most amygdalar regions (green arrows), indicating astrocytes in these regions originate from *Nkx2.1*^*-*^ PD. **B**, Schematics illustrating the association of neuron-astrocyte and astrocyte-astrocyte originating from distinct PDs. Essentially, the distribution of neurons and astrocytes depends on PDs, however, in certain regions such as the striatum and piriform cortex, neurons and astrocytes originating from distinct PDs are closely associated (circled region). This might be due to across-region tangential migration of neurons (dotted arrows). Although the regions of astrocyte distribution were generally segregated from those originating from distinct PDs, astrocytes from distinct domains were intermingled in certain regions such as the ventral part of the striatum and olfactory tubercle (region shown by rectangle).

Using the method of IUE of plasmids, which allows integration of transgene into the genome, we visualized descendants originating from progenitors in the subpallium. Labeled cells were found in the neocortex, hippocampus, olfactory bulb, olfactory tubercle, nucleus accumbens, piriform cortex, striatum and amygdala. Although both neurons and astrocytes were labeled in many brain regions, virtually no astrocytes were labeled in the neocortex, hippocampus, olfactory bulb or nucleus accumbens of IUE performed animals (Fig. 7A). Likewise, neurons were almost exclusively labeled in the neocortex, hippocampus, dorsal part of the striatum, piriform cortex and most subnuclei of the amygdala of *Nkx2.1*^*Cre*^*; Ai9* mice (Fig. 7A). These results suggest that there exist neurons that do not associate with astrocytes originating from the PD of their own. Indeed, IUE on *Nkx2.1*^*Cre*^*; Ai9* animals revealed that some *Nkx2.1*^*+*^ PD-derived neurons were closely associated with astrocytes from *Nkx2.1*^*–*^ PDs. We also found that astrocytes from distinct PDs were closely associated. These findings suggest that neurons as well as astrocytes in the forebrain can be associated with astrocytes originating from PDs distinct from their own.

Observation of labeled cells in mature brains does not allow us to identify the domains of labeled progenitors by IUE. However, we found that GEs were the major site of labeling by examining E12.5–14.5 embryos (Fig. 1). Our findings that cortical interneurons were labeled by IUE are consistent with the results of genetic fate-mapping^26^,^36^,^41^,^44^,^47^, and transplantation studies^19^,^20^, which have shown that the MGE, CGE and POA are the major sites of cortical interneuron generation. It has also been shown that GEs give rise to the cells in the olfactory bulb, olfactory tubercle, piriform cortex, striatum, nucleus accumbens, globus pallidus and amygdala^19^,^20^,^41^, which is consistent with our results.

We have shown massive generation of astrocytes in addition to neurons in the subpallium. Consistently, a recent study in which *Nkx2.1*^*CreERT2*^was crossed with a *Rosa*^*tdTomato*^ reporter line and conditionally caused recombination at E17 reported the generation of astrocytes in the ventromedial forebrain^4^. In the present study, we analyzed the distribution of labeled astrocytes in the forebrain of IUE-labeled animals in detail and showed that VZ progenitors in the subpallium at E10.5 give rise to astrocytes in the olfactory tubercle, ventral part of the striatum, globus pallidus, amygdala and lateral septal nucleus. Additionally, our IUE experiments showed that *Nkx2.1*^*–*^ PDs in the subpallium also give rise to astrocytes in the olfactory tubercle, striatum, piriform cortex and amygdala.

The Tol-2 system has been successfully used to track VZ progenitors descendants in the cerebellum^24^ and cerebral cortex^48^. Application of this method allowed us to label glial cells in addition to neurons in these previous studies. The present finding that astrocytes were labeled only by these methods but not by conventional plasmids, which is not integrated into the genome (Torigoe *et al.*, unpublished observation), indicates that astrocytes were generated after the repeated division of progenitors, which agrees with the case of the cortical astrocytes^14^,^48^,^49^ and cerebellar astrocytes^24^.

Both neurons and astrocytes were labeled in the olfactory tubercle, striatum, piriform cortex and amygdala by IUE. However, virtually no astrocytes were labeled in the dorsal striatum, piriform cortex or most of the amygdalar regions in *Nkx2.1*^*Cre*^*; Ai9* animals (Fig. 7A, dotted green arrows), although quite a few astrocytes were labeled in the olfactory tubercle, ventral part of the striatum, nucleus accumbens and lateral septal nucleus (Fig. 7A, red arrows). This finding suggests that *Nkx2.1*^+^ PDs give rise to astrocytes only in restricted regions of the subpallium. Given that IUE-labeled (GFP^+^) but not *Nkx2.1*^*Cre*^*; Ai9*-labeled (tdTomato^+^) astrocytes were densely labeled in the dorsal striatum, piriform cortex and amygdala, it is likely that astrocytes in these regions originated from progenitors of *Nkx2.1*^–^ PDs such as the LGE, CGE and ventral pallium.

We found neurons in the ventral forebrain were associated with astrocytes originating from the same PD, but those in the neocortex, hippocampus, olfactory bulb and nucleus accumbens were not. Similar findings were previously reported for interneurons in the neocortex^13^. One possible reason for the absence of labeled astrocytes might be the lack of astrocyte capacity to migrate tangentially. Cortical interneurons, hippocampal interneurons and olfactory bulb granule/periglomerular cells reach their final position after extensive tangential migration^50^. Thus, a likely possibility is that neurons that migrate tangentially are not associated with astrocytes with common PD, although other possibilities such as selective death of dorsally migrating astrocytes cannot be excluded.

Both neurons and astrocytes were labeled in the olfactory tubercle, piriform cortex, striatum and amygdala by IUE. However, the distribution of astrocytes was not uniform within these regions. In the striatum, for example, only neurons were labeled in the rostral part in some preparations, while the caudal part was always associated with astrocytes. A local distribution of astrocytes was also found in *Nkx2.1*^*Cre*^; *Ai9* mice. Astrocytes were present only in the ventral part of the striatum and only in the CA and MA of the amygdala. It remains unknown how this localized distribution of astrocytes is achieved. It is conceivable that astrocyte proliferation^51^ or death is differentially regulated between different subregions. An alternative possibility is a difference in migratory capacity of neurons or astrocytes. Although the exact pattern of migration of neurons in the subpallium is not well documented, there is evidence that they migrate tangentially after executing radial migration^52^. Thus, neurons that have migrated tangentially may not be associated with astrocytes. The validity of these ideas requires future studies.

Although astrocytes and neurons originating from a common PD appear to interact in the spinal cord^4^,^7^, our finding that neurons but not astrocytes were labeled in certain regions suggests that neurons in such regions might interact with astrocytes from distinct PDs (Fig. 7B). Consistent with this notion, IUE-labeled astrocytes displayed hole-like structures that were occupied by neurons (Fig. 6F). More importantly, the present study directly demonstrated the association of astrocytes and neuron from distinct PDs. We showed that neurons of *Nkx2.1*^*+*^ progenitor origin were in close apposition with astrocytes from *Nkx2.1*^–^ PDs. The fact that tdTomato^–^ hole-like structures were found for tdTomato^+^ astrocytes in *Nkx2.1*^*Cre*^*; Ai9* mice (Fig. 5N, red arrow and 6G2, white arrow) suggests that neurons originating from PDs other than the *Nkx2.1*^*+*^ PD are located there. Collectively, these findings indicate that at least a subset of neurons in the forebrain are associated with astrocytes from a distinct PD (Fig. 7B).

We have also found that astrocytes from distinct PDs co-existed in the ventral striatum and olfactory tubercle (Fig. 6G,I). This indicates that astrocytes in these regions are not necessarily homogeneous. It would be interesting to know whether astrocytes from different PDs contact different types of neurons.

Curiously, the processes astrocytes from the *Nkx2.1*^*+*^ PDs did not invade into the territory occupied by processes of astrocytes from distinct PDs, forming a mosaic-like pattern. Previously, it was shown that neighboring astrocytes in the hippocampal CA1 region establish primarily exclusive territories^46^. It remains unknown whether these neighboring astrocytes in the hippocampus have distinct PD origins or not. In any case, it would be interesting to explore if astrocytes from distinct PDs generally establish primarily exclusive territories.

Although the interaction of neurons with astrocytes of a common developmental origin appears to be important for neuronal development and function in the spinal cord, the present study suggests that this may not be the case for some types of neurons in the forebrain. On the contrary, our results raised the possibility that astrocytes and neurons originating from distinct PDs can interact with each other.

## Materials and Methods

### Animals

The following mice were used: ICR wild type (Nihon SLC), *Nkx2.1*^*Cre*^ mice (Stock no. 008661, Jackson Laboratory) and *Ai9* mice (Stock no. 007905, Jackson Laboratory). These transgenic mice were backcrossed onto the ICR background. *Nkx2.1*^*Cre*^ mice were crossed with *Ai9* mice to fate map *Nkx2.1*^*+*^ progenitors. The noon of vaginal plug detection was designated as embryonic day 0.5 (E0.5). The day of birth was designated as postnatal day 0 (P0).

EdU or BrdU was intraperitoneally injected at E10.5, E12.5, E14.5 or E16.5 in some of the animals. Although these injections were made for another purpose, data from these animals were also included in the analysis. We found no difference in gross anatomy or in the distribution of labeled cells between injected and un-injected animals.

All experiments involving mouse care, surgery and sample preparation were approved by the Animal Experimental Committee of Osaka University Graduate School of Frontier Biosciences and performed in accordance with the NIH guidelines and the Osaka University Guidelines for the Welfare and Use of Laboratory Animals.

### Preparation of tissue sections

After immobilization by cold anesthesia, the brains of embryos were dissected in cold phosphate-buffered saline (PBS) and immersed in 4% paraformaldehyde (PFA) in 0.12 M phosphate buffer (PB) overnight at 4 °C. Adult (P21-30) mice were deeply anesthetized with sodium pentobarbital (Kyoritsu Pharmacy; 100 mg/kg body weight) and perfused transcardially with PBS followed by 4% PFA in 0.12 M PB and immersed overnight in the same fixative at 4 °C. The brains were cryoprotected by immersion in 30% sucrose in 0.1 M PB overnight, embedded in OCT compound (Sakura Finetek) and quickly frozen in liquid-nitrogen cooled isopentane. For embryos, transverse sections were cut on a cryostat (HM500, Zeiss) at 20 or 30 μm and the sections that included ganglionic eminences (GEs) were mounted on slides (Superfrost Plus, Fisher Scientific). For adult mice, serial sections were cut coronally at 50 μm and 80–100 sections were cut rostrocaudally starting from the caudal end of the rhinal incisure. In some animals, sections were cut from the level of the olfactory bulb. Every ten sections were then collected as free-floating sections, subjected to immunostaining and mounted on slides.

### Immunohistochemistry

Immunostaining was carried out essentially as described previously^53^,^54^,^55^. The following primary antibodies were used: goat polyclonal anti-aldolase C (1:50, sc-12066, Santa Cruz Biotechnology), rabbit polyclonal anti-Dsred (1:300, 632496, Clontech), mouse monoclonal anti-glutamate decarboxylase-67 (GAD67) (1:4000, MAB5406, Merck Millipore), chicken polyclonal anti-green fluorescent protein (GFP) (1:1000, ab13970, Abcam), rat monoclonal anti-GFP (1:1000, 04404–84, Nacalai Tesque), mouse monoclonal anti-NeuN (1:50, MAB377, Millopore), rabbit monoclonal anti-Nkx2.1 (1:2000, ab76013, Abcam) and rabbit polyclonal anti-S100 beta (1:500, ab868, Abcam).

The following secondary antibodies were used: Alexa Fluor 488-conjugated donkey anti-chicken IgY (1:500, 703–545–155, Jackson ImmunoResearch), Alexa Fluor 488-conjugated goat anti-chicken I gY (1:500, 103–545–155, Jackson ImmunoResearch), Alexa Fluor 488-conjugated donkey anti-rat I gG (1:500, A21208, Life Technologies), Alexa Flour 594-conjugated goat anti-rabbit I gG (1:1000, A11012, Molecular Probes), Alexa Fluor 647-conjugated donkey anti-rabbit I gG (1:500, A31573, Life Technologies), Alexa Fluor 647-conjugated goat anti-rat I gG (1:500, A21247, Invitrogen), Cyanine 3 (Cy3)-conjugated donkey anti-mouse IgG (1:500, 715–166–151, Jackson ImmunoResearch), Cy3-conjugated goat anti-rat I gG (1:500, 612–104–120, Rockland Immunochemicals) and Cy5-conjugated donkey anti-mouse IgG (1:500, 715–175-150, Jackson ImmunoResearch).

All antibodies were diluted in 0.5% (0.2% in the case of embryos) Triton X-100 in PBS with 1% normal goat serum or normal horse serum.

### IUE

GE-directed IUE was performed on E10.5 wild type, *Ai9* reporter mice and *Nxk2.1*^*Cre*^*; Ai9* mice as described^17^,^56^ with some modifications. Five electric pulses (27 V for 50 ms) were delivered at 950 ms intervals with forceps-shaped electrodes (CUY650P2, Unique Medical Imada) connected to an electroporator (CUY21, Nepa Gene).

To achieve labeling of all progenitor descendants, the Tol-2 system was used^57^. For this purpose, 1–2 μg/μl *pCAGGS*^58^-*Tol2 transposase (T2TP)* and 1 μg/μl *pT2K-CAGGS*-*enhanced green fluorescent protein (EGFP)*^22^ were co-electorporated with 1 μg/μl *pCAGGS-tandem dimer tomato (tdTomato)* or *monomeric cherry (mCherry)*^59^ to wild type and 1–2 μg/μl *pCAGGS-T2TP* and 1 μg/μl *pT2K-CAGGS-EGFP* to *Nkx2.1*^*Cre*^; *Ai9* mice. As a second method to label all progenitor descendants, 1 μg /μl *pCAGGS-Cre* and 1 μg/μl *pCAGGS-EGFP*^60^ were co-electroporated to *Ai9* mice embryos. In either method, plasmids were dissolved in PBS.

In all samples, GFP and tdTomato fluorescence were enhanced by immunostaining against GFP and DsRed, respectively.

### Image Processing

Images were acquired using a Leica confocal microscope (TCS SP5 AOBS; Leica Microsystem). Z-series stacks of confocal images were acquired at 7.0, 2.5, 1.2 or 0.5 μm intervals using a 10x (N.A., 0.4), 20x (N.A., 0.7), 40x (N.A., 0.85) or 63x (N.A. 1.4) objective lens, respectively, at 1024 × 1024 pixel resolution with a pinhole setting of one Airy unit. Acquired images were tiled, stitched and processed to make maximum projection images using a confocal software (LAS AF Ver. 2.00, Leica). Images were processed with Adobe Photoshop (CS3, 5.1 or 6) and assembled with Adobe Illustrator (CS3 or CS6) (Adobe systems Inc., CA, USA).

### Labeled cells in the diencephalon

In many cases in which IUE was performed, we found labeled cells in the zona incerta and thalamic reticular nucleus. Likewise, these structures were also labeled in *Nkx2.1*^*Cre*^*; Ai9* animals. Although these cells might have originated from ganglionic eminences, the possibility that they originated from IUE-labeled or *Nkx2.1*-expressing diencephalic structures cannot be precluded. Therefore, labeled cells in the thalamus and the hypothalamus were excluded from the analysis.

## Additional Information

**How to cite this article**: Torigoe, M. *et al.* Association of astrocytes with neurons and astrocytes derived from distinct progenitor domains in the subpallium. *Sci. Rep.* **5**, 12258; doi: 10.1038/srep12258 (2015).

## Supplementary Material

Supplementary Information

## Figures and Tables

**Table 1:** Summary of the labeled sites in embryos.

**Site of EP**	**E12.5**						**E13.5**		**E14.5**
	**1**	**2**	**3**	**4**	**5**	**6**	**7**	**8**	**9**
LGE	++	++	++	+	+	+	++	+	+
MGE	+	−	+	++	+	−	++	−	−
CGE	+	+	+	−	+	−	++	+	+
VP	+	+	+	+	−	+	−	+	+
POA	−	−	+	+	−	−	−	−	−

Transverse sections were cut in embryos 2–4 days after IUE. LGE: lateral ganglionic eminence, MGE: medial ganglionic eminence, CGE: caudal ganglionic eminence. VP: ventral pallium. POA: preoptic area. ++ indicates labeling of the entire cross sectional area. + represents partial labeling of the cross sectional area.
