# Supplementary material for: Association of astrocytes with neurons and astrocytes derived from distinct progenitor domains in the subpallium
Source: Sci Rep. 2015 Jul 20;5:12258. doi: 10.1038/srep12258 (PMC4648416; doi:10.1038/srep12258)
Supplement: Supplementary Information [file srep12258-s1.pdf]

Association of astrocytes with neurons and astrocytes derived from distinct progenitor domains in the subpallium

Makio Torigoe[1,2], Kenta Yamauchi[1,3], Yan Zhu[1,3], Hiroaki Kobayashi[1] and

*Fujio Murakami[1]

[1]Graduate School of Frontier Biosciences, Osaka University, Suita, Osaka 560-8531, Japan,

Present address: [2]Lab. Dev Gene Regulation, RIKEN, BSI, 2-1 Hirosawa, Wako, 351-0198, Saitama, Japan

[3] Division of Brain Function, National Institute of Genetics, 1111 Yata, Mishima 411-8540, Shizuoka, Japan

Corresponding author :Fujio Murakami

Graduate School of Frontier Biosciences, Osaka University, Suita, Osaka 560-8531, Japan,

E-mail:murakami@fbs.osaka-u.ac.jp

Phone: +81-6-6879-4655/050-3639-3519, Fax: +81-20-4622-5947

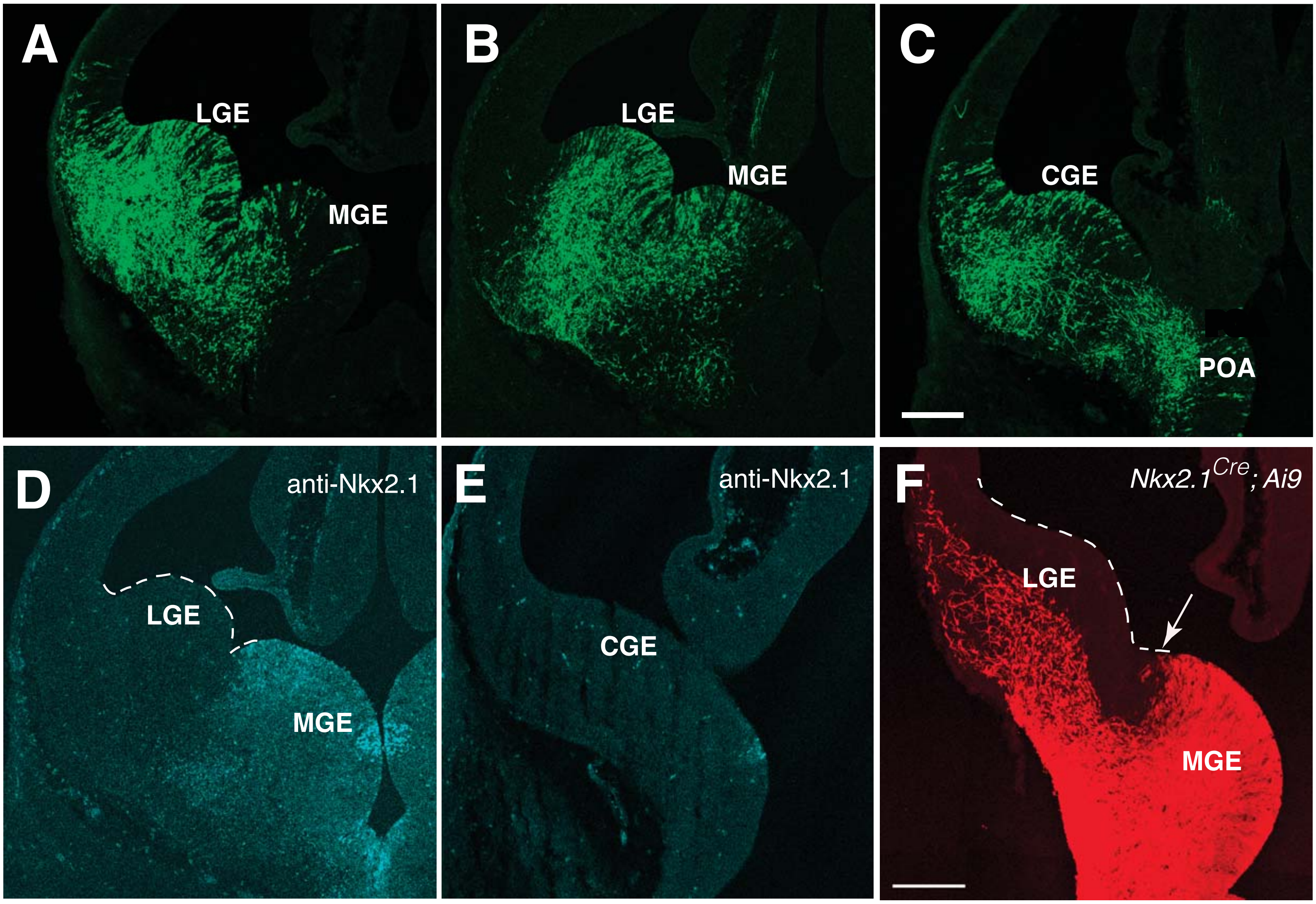

Supplementary Figure S1

Legend to supplementary Figures S1

Labeled sites by IUE. A-E show transverse sections of an E12.5 forebrain 2 days after electroporation. LGE is predominantly labeled in A, both MGE and LGE are labeled in B and CGE is predominantly labeled in C. D-E, Immunostaining for Nkx2.1. Nkx2.1 is specifically expressed in the MGE, but not the LGE (D) and the CGE (E). Tol-2 system was used for electroporation. F, E12.5 forebrain of an *Nkx2.1^{Cre}; Ai9* mouse. TdTomato is expressed in the ventricular zone of the MGE except in its dorsalmost part (arrow), but not in the LGE. LGE: lateral ganglionic eminence, MGE: medial ganglionic eminence, CGE: caudal ganglionic eminence. POA: preoptic area. All scale bars are 200 μm and that in C applies to A-C, that in D to D-E.

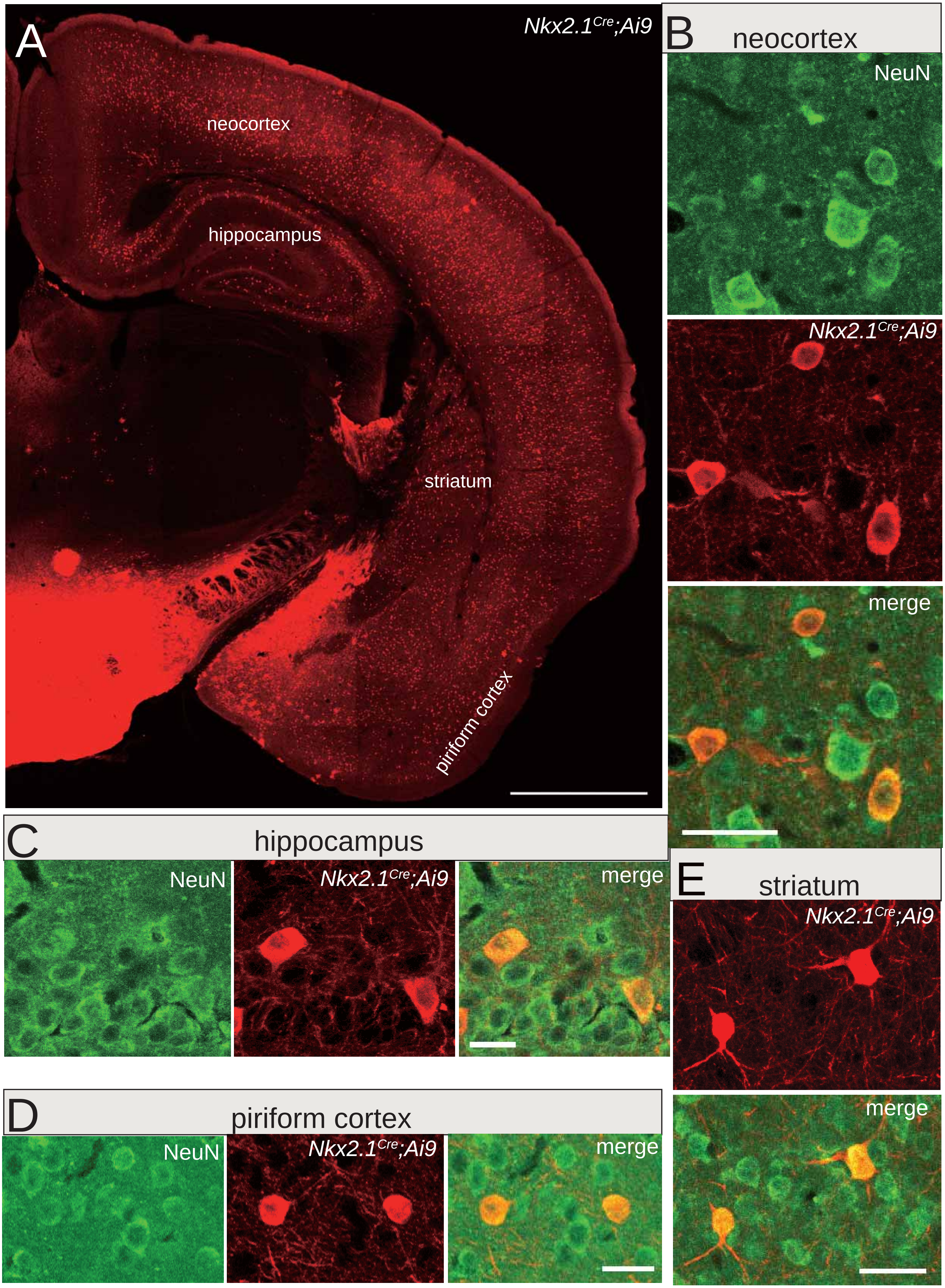

Supplementary Figure S2

Legend to supplementary Figures S2

Neuronal nature of *Nkx2.1*^{Cre}*; Ai9* fate-mapped cells. A, Coronal section at the level that includes the striatum and hippocampus. B-E, High-magnification views of *Nkx2.1*^{Cre}*; Ai9* fate-mapped cells in the neocortex (B), hippocampus (C), piriform cortex (D) and striatum (E). In B - E, green signals represent NeuN expression and red signals represent tdTomato expression of *Nkx2.1*^{Cre}*; Ai9* fate-mapped cells. Lowermost or rightmost panels are merged views. Scale bar: 1 mm in A, 30 µm in B and E, and 20 µm in C and D.

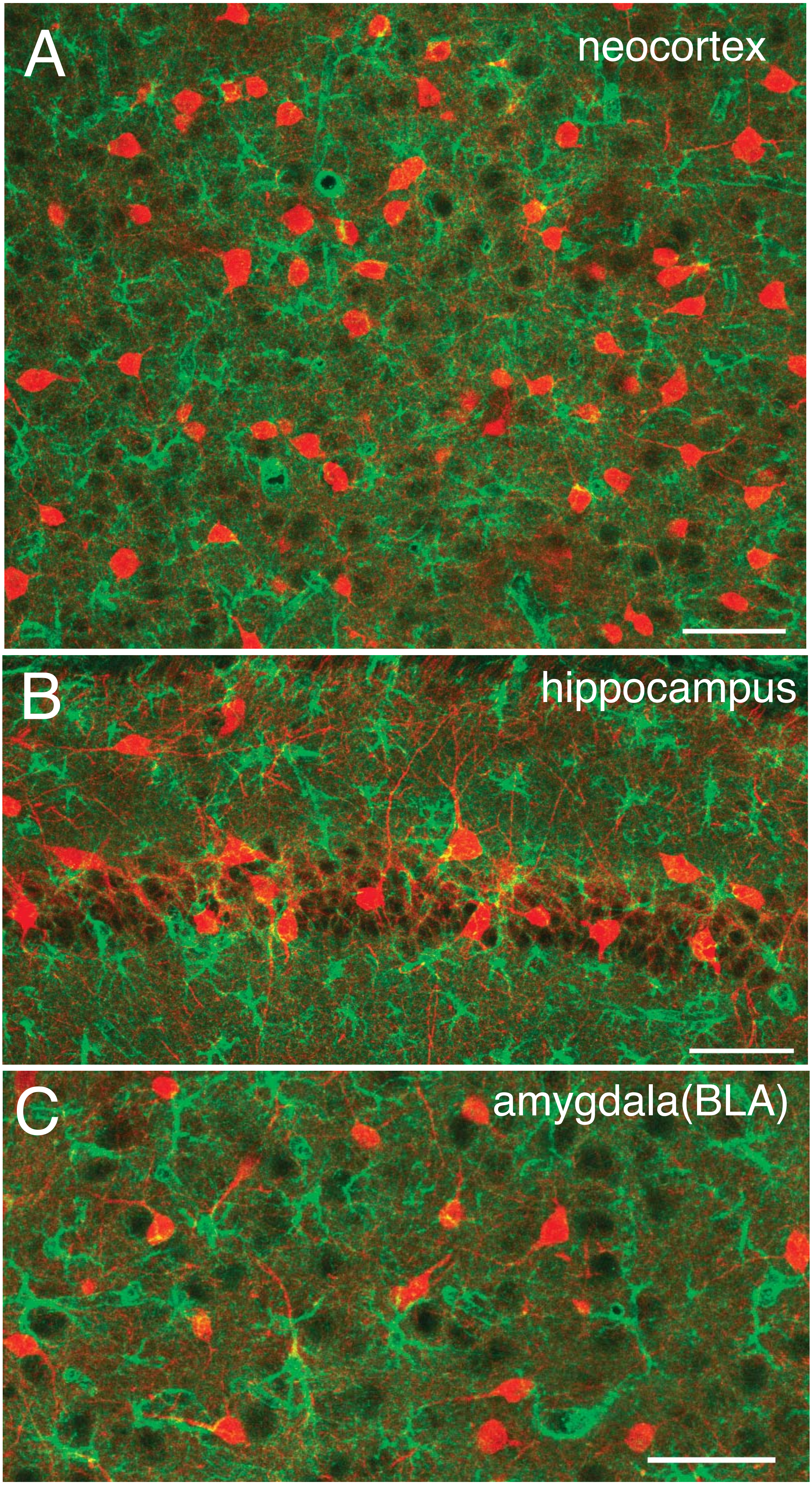

Supplementary Figure S3

Legend to supplementary Figures S3

Segregation of *Nkx2.1*^{Cre}*; Ai9* fate-mapped cells from signals of an astrocyte marker, Aldolase C, in several forebrain regions. Images acquired from the neocortex (A), hippocampus (B) and basolateral complex of the amygdala (C). Merged views of *Nkx2.1*^{Cre}*; Ai9* fate-mapped cells (red) and immunostained processes of astrocytes by anti Aldolase C (green). *Nkx2.1*^{Cre}*; Ai9* fate-mapped cells do not possess astrocytic processes. Scale bars: 50 μm.

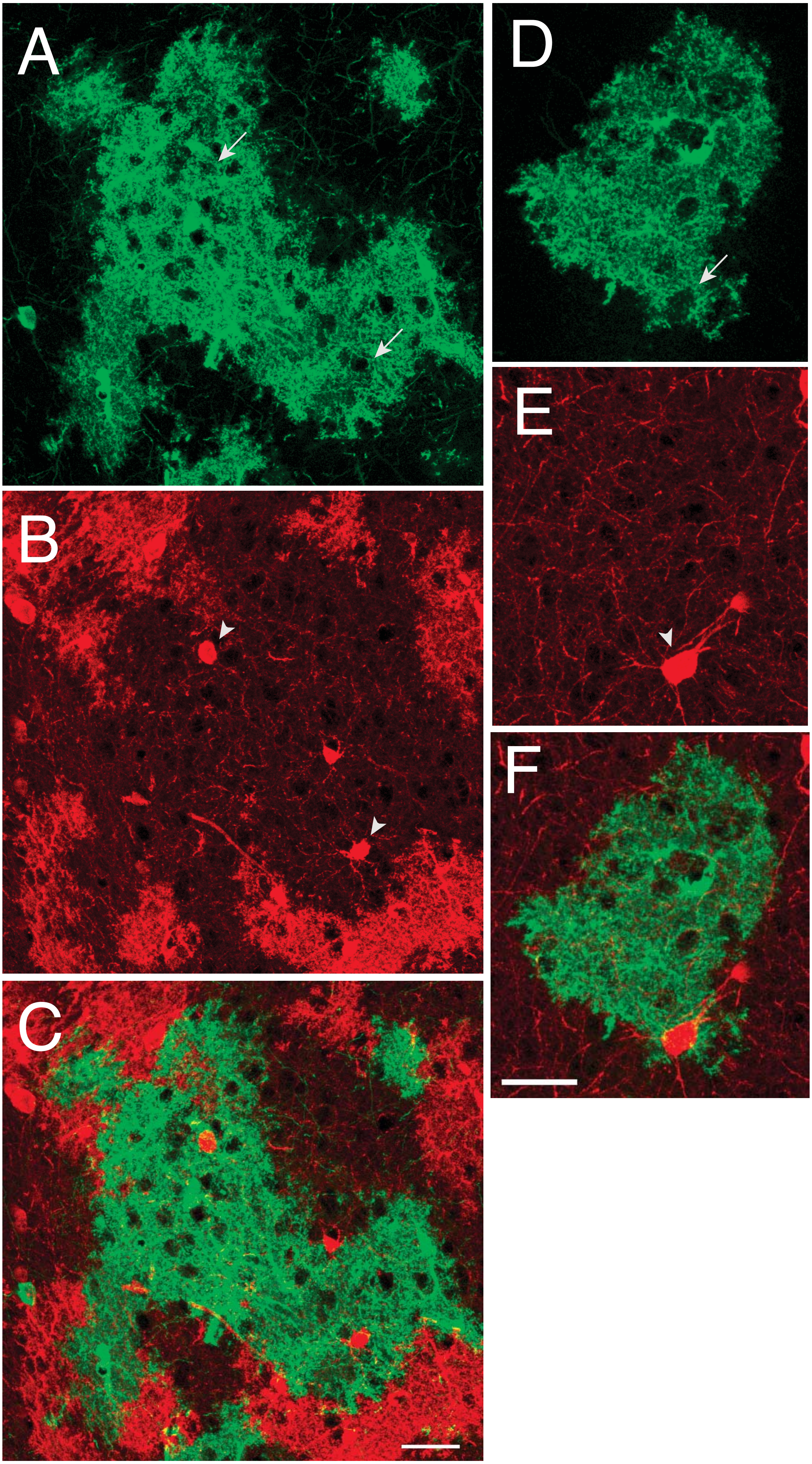

Supplementary Figure S4

Legend to supplementary Figures S4

Association of neurons and astrocytes from distinct PDs. IUE was performed on *Nkx2.1*^{Cre}*; Ai9* animals. A-C are images taken from the olfactory tubercle and D-F are those from the striatum. A and D are GFP-positive astrocytes labeled by IUE, and B and E are *Nkx2.1*^{Cre}*; Ai9* fate-mapped cells. C and F are merged views. Arrows in A and D point to the positions of neurons shown in B and E (arrowheads). A-C and D-F were captured from two different animals. Both of them are different from the animal shown in Figure 6. Scale bars: 30 μm
